# A Systematic Review of the Guidelines on Venous Thromboembolism Prophylaxis in Gynecologic Oncology

**DOI:** 10.3390/cancers14102439

**Published:** 2022-05-15

**Authors:** Federico Romano, Giovanni Di Lorenzo, Guglielmo Stabile, Mariateresa Mirandola, Stefano Restaino, Patrizia Ianniello, Giuseppe Mirenda, Giuseppe Ricci

**Affiliations:** 1 Institute for Maternal and Child Health, IRCCS Burlo Garofolo, Via dell’ Istria 65/1, 34137 Trieste, Italy; federico.romano@burlo.trieste.it (F.R.); giovanni.dilorenzo@burlo.trieste.it (G.D.L.); giuseppe.mirenda@burlo.trieste.it (G.M.); giuseppe.ricci@burlo.trieste.it (G.R.); 2 Department of Medicine, Surgery and Health Sciences, University of Trieste, Strada di Fiume 447, 34127 Trieste, Italy; tea.mirandola@gmail.com (M.M.); patrizia.ianniello@burlo.trieste.it (P.I.); 3 Department of Maternal and Child Health, University-Hospital of Udine, P.le S. Maria della Misericordia 15, 33100 Udine, Italy; restaino.stefano@gmail.com

**Keywords:** venous thromboembolism prevention, thromboprophylaxis, gynecological cancer, deep vein thrombosis, pulmonary embolism

## Abstract

**Simple Summary:**

Cancer-associated venous thromboembolism (VTE) is the second leading cause of death in cancer patients. Gynecological cancer patients are considered at high risk of VTE and of bleeding due to the intrinsic nature of the tumor itself. Prevention of VTE in this special subgroup of cancer patients is a current topic of interest since there are no standardized protocols. This review aimed to summarize the protocols for VTE prevention in gynecological cancer patients given by the selected national and international guidelines in order to help the clinicians to identify patients who would benefit from VTE prophylaxis during daily practice.

**Abstract:**

(1) Background: This review aimed to summarize the indications for venous thromboembolic (VTE) events’ prophylaxis in a gynecological cancer population, according to the most recent guidelines. (2) Methods: A systematic review of the guidelines in PubMed, SCOPUS, Web of Science, EMBASE, and CINHAL regarding VTE prevention in gynecological cancer patients was conducted according to PRISMA criteria. We compared the recommendations given by oncological and hematological societies regarding VTE prevention in gynecological cancer patients published from January 2010 through March 2021. We searched for the following keywords: “venous thromboembolism prevention”, “cancer”, and “guidelines”. The AGREE II checklist was used to critically analyze the guidelines’ quality. (3) Results: There were 1003 documents available; 14 met the inclusion criteria, 5 were excluded and, eventually, the guidelines of 10 societies were evaluated. (4) Conclusions: The guidelines agree that low-molecular-weight heparin (LMWH) and fondaparinux achieve better results in VTE prevention in gynecological cancer patients. Direct oral anticoagulants (DOACs) can be used to prevent VTE in outpatients and high-risk medical patients after discharge. VTE risk scores should be applied to all oncological patients to identify those who would benefit from a prevention program. More attention should be paid to mechanical prophylactic methods due to the high bleeding risk of gynecological cancer patients.

## 1. Introduction

The main cause of death in middle- and high-income world regions is represented by cardiovascular diseases, as the World Health Organization (WHO) indicated. In developed countries, venous thromboembolic (VTEs) events ARE considered the third most common cardiovascular disease after coronary heart disease and stroke, with one case per 1000 inhabitants per year [1]. For the first time in 1856, Virchow defined venous thrombosis predisposing factors based on a triad of events: blood stasis, vascular damage, and hypercoagulability [2,3].

Cancer-associated thrombosis incidence is increasing worldwide, and it represents the second leading cause of death in cancer patients [4], often complicating the clinical course and delaying therapy [5,6]. These patients are significantly more likely to develop VTE (10–25% of cancer patients) and experience higher rates of VTE recurrence and bleeding complications during antithrombotic treatment [7].

Many studies demonstrated that there is an association between thromboembolism and tumor, endorsing the hypothesis that the last one promoted a prothrombotic state [8,9,10]. Indeed, cancer cells can stimulate the coagulation cascade by a direct mechanism, producing procoagulant substances such as “tissue factor” or “cancer procoagulant,” and through indirect mechanisms, activating blood cells, such as monocytes, platelets, and endothelial cells, inducing the expression of a procoagulant phenotype in these cells [5,6]. These factors join specific cancer complications such as tumor compression stasis, the presence of an inflammatory state, dysproteinemia, supervening infections, and immobility. Finally, the prothrombotic effect can be caused by chemotherapy and the devices used to infuse drugs such as central venous catheters (CVCs) [11].

Gynecological cancer subjects were reported as having a 4.2% occurrence of VTE compared with a 0.2% in non-oncological ones, all of them without medical prophylaxis [12,13].

VTE in cancer patients is more likely to be associated with specific tumors, of which gynecological, pancreatic, and stomach cancers are considered the most frequently associated [14,15,16].

Specific characteristics of each type of gynecological cancers are described to be implicated in a more significant risk of VTE. The ovarian cancer population has more chances to experience VTE than other gynecological tumor types [17]. In the context of cervical cancer, the dimension of the tumor is correlated with an increased risk of VTE: the >5 cm tumor size has a nine-fold increased risk (10% vs. 1.2%) [15]. Regarding endometrial cancer subjects, VTE incidence varies depending on tumor histology: endometrioid grade 3 histologies are associated with an increased prospect of a 6-month VTE incidence compared with low-grade histologies [18].

Oncological treatment, chemotherapy, and radiotherapy, in addition to surgical treatment, often worsen VTE incidence. Moreover, when VTE complications occur, patients’ mortality increases [19].

Furthermore, patients affected by gynecological malignancies, especially in advanced stages, are likely to suffer from bleeding sequelae due to the nature of the tumor itself, which could complicate the management of VTE prophylaxis. An earlier systematic review and meta-analysis of gynecologic surgery patients suggested that while pharmacological prophylaxis decreases the risk of VTE by approximately 50%, it leads to an increase in the risk of major postoperative bleeding by a similar percentage [20]. The risks also vary based on the specific site of the neoplasm and surgical procedure performed. In patients who underwent a minimally invasive surgery, the overall VTE rates were significantly lower. However, the magnitude of this variation is uncertain [21]. The management of these patients is, therefore, based on the delicate balance between VTE and cancer-associated bleeding risk. The specific risk assessment of VTE cannot be underestimated when dealing with gynecological cancer patients.

This work aimed to summarize and compare the indications of the international societies of oncology and hematology on VTE primary prevention in hospitalized and ambulatory gynecological cancer patients, both in surgical and non-surgical settings.

## 2. Materials and Methods

### 2.1. Search Strategy and Selection Criteria

We performed a systematic review of the guidelines present in PubMed, SCOPUS, WOS, EMBASE, and CINHAL regarding VTE prevention in gynecological cancer patients.

The articles’ research was performed following the Preferred Reporting Items for Systematic Reviews and Meta-Analyses [20].

The terms “venous thromboembolism prevention”, “cancer”, and “guidelines” were selected for the search in the databases mentioned above. We carefully chose those guidelines that focused on VTE prevention in gynecological oncological patients. The research included publications from January 2010 to December 2021.

We included articles potentially relevant and related to the topic, written in English or Italian by oncological and hematological scientific societies. Studies that were not guidelines and not pertinent to the topic were excluded. A narrative description of the findings was conducted. No statistical analysis or meta-analysis was performed.

### 2.2. Data Extraction and Checking

Two reviewers (M.M. and G.R.) independently searched in the above-listed databases and extracted the published data. Unresolved questions and disagreements were resolved by an open discussion with the other authors.

### 2.3. Quality Assessment

The AGREE II (Appraisal of Guidelines for Research and Evaluation) reporting checklist was employed to appraise guidelines critically [21]. This tool is the most widely used for assessing guideline quality [22]. The appraiser is requested to rate each of the 23 items proposed based on a scale from 1 (strongly disagree) to 7 (strongly agree). The 23 items address six domains ((1) Scope and purpose; (2) Stakeholder involvement; (3) Rigor of development; (4) Clarity of presentation; (5) Applicability; and (6) Editorial independence). The web-based platform My AGREE PLUS (https://www.agreetrust.org/my-agree/, accessed on 10 August 2021) was used to complete appraisals online, based on the user manual (available at the following link: http://www.agreetrust.org/, accessed on 10 August 2021). The score for each domain is calculated by summing the scores of the individual items in the domain and calculating the percentage value of the maximum possible score; the same procedure is performed for the overall score. Domain and overall scores below 50–60% are considered to be low-quality indicators of the guideline analyzed [23]. All the included guidelines in the present paper scored more than 60% (Table 1).

## 3. Results

**Figure 1:**
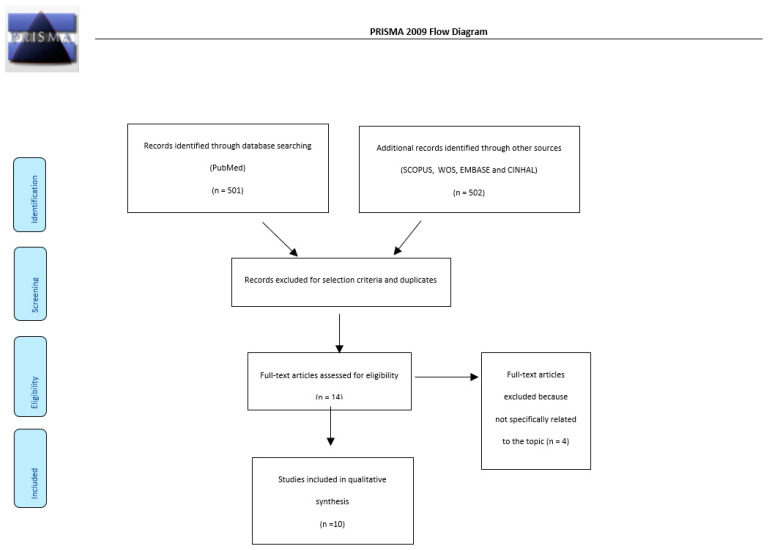
Prisma flow diagram of our review.

After a crossmatch search, 1003 articles were selected; 14 were considered eligible and 4 were excluded because they were not guidelines, they were duplicates, or they were not precisely relevant to the topic. Eventually, 10 guidelines were judged as suitable for analysis (Figure 1).

We examined each guideline and summarized the main recommendations (Table 2). We compared different recommendations in order to define optimal and univocal management of VTE prevention in cancer patients.

The guidelines included in the systematic review were:

- The National Comprehensive Cancer Network (NCCN), 2020 [24];
- The American Society of Clinical Oncology (ASCO), 2020 [25];
- The International Initiative on Thrombosis and Cancer and the International Society on Thrombosis and Haemostasis (ITAC/ISTH) with the support of the French National Cancer Institute, 2019 [26];
- The Italian Association of Medical Oncology (AIOM) 2019 [27];
- The Spanish Society in Medical Oncology (SEOM) clinical guideline of venous thromboembolism and cancer, 2019 [28];
- The Haemostasis and Thrombosis Task Force of the British Committee for Standards in Haematology (BCSH), 2015 [29];
- The Canadian consensus recommendations on the treatment of VTE in cancer patients, 2015 [30];
- The European Society of Medical Oncology (ESMO) 2011 [31];
- The Italian Society for Haemostasis and Thrombosis (SISET), 2011 [32].
- The American Society of Hematology (ASH), 2021 [33]

Almost all guidelines reported the Khorana score, which was introduced in 2008 [34] (Table 3) to evaluate the patient’s VTE risk periodically.

### 3.1. National Comprehensive Cancer Network (NCCN)

NCCN Guidelines discussed diagnosis, prevention, and treatment of VTE in cancer patients and provided recommendations for patient care based on clinical research and experience in this field.

The latest version of 2020 focused on different aspects of VTE in cancer patients, basically giving indications for VTE prevention in out- and inpatients.

Personalized medicine was pursued through the detailed description of bleeding/thrombosis risk according to Khorana’s score [34] (Table 3) and other risk evaluation flow charts (Supplementary Tables S1–S3). Contraindications and warnings for the use of anticoagulant drugs were synthesized in a useful chart (Supplementary Table S4).

Concerning VTE prophylaxis, the guideline recommended:

- UFH OR LMWH prophylaxis in all oncological inpatients;
- Post-surgical prophylaxis for at least 4 weeks;
- Intermittent pneumatic compression (IPC) was suggested for inpatients with cancer not suitable for pharmacological prophylaxis;
- Fondaparinux could be safely used;
- DOACs (apixaban and rivaroxaban) were suggested for discharged medical patients and outpatients under chemotherapy following the Khorana score, from 3 to 6 months;
- CVC patients should not be routinely treated with anticoagulant prophylaxis unless they were at high risk for VTE.

### 3.2. The American Society of Clinical Oncology (ASCO)

ASCO published for the first time an evidence-based clinical practice guideline on prophylaxis and treatment of VTE in 2007. The guidelines were regularly updated at intervals determined by an Update Committee; the last update was in 2020 and focused primarily on the efficacy and safety of DOACs in the prevention of VTE in cancer patients. DOACs were suggested to be safely used as an alternative to LMWH in preventing VTE in patients at risk but should be carefully advised in selected subjects with low gastrointestinal tract bleeding risk. In particular, DOACs were suggested to prevent VTE in high-risk patients receiving chemotherapy and high-risk non-surgical patients after discharge. ASCO guidelines suggested adopting the Khorana score [34] (Table 3) to establish the risk of VTE in these patients. LMWH anticoagulant prophylaxis should be offered to all hospitalized cancer patients and after surgery (up to 4 weeks in major procedures). Inferior vena cava filters could be offered to patients with absolute contraindications to anticoagulant drugs only in the acute treatment setting; therefore, it was not indicated with prophylactic intentions.

### 3.3. The International Initiative on Thrombosis and Cancer and the International Society on Thrombosis and Haemostasis (ITAC/ISTH), with the Support of the French National Cancer Institute

The International Initiative on Thrombosis and Cancer is an independent academic working group that aimed to establish a global consensus for the treatment and prophylaxis of VTE in patients with cancer. The 2019 clinical practice guidelines were based on a systematic review of the literature published up to December 2018 and were presented with a Grading of Recommendations, Assessment, Development, and Evaluation scale methods, with the support of the French National Cancer Institute. These guidelines were reviewed by an expanded international advisory committee and endorsed by the International Society on Thrombosis and Haemostasis.

According to the guidelines, prophylaxis should be given to all surgical cancer patients for at least 7 days (up to 4 weeks in major abdominal–pelvic surgery) and only to ambulatory outpatients at risk.

Different types of VTE assessment risks were listed in this guideline: Khorana score [34] (Table 3), COMPASS-CAT score [35] (Table 4), and ONKOTEV score [36] (Table 5).

Interestingly, VTE prophylaxis was not strongly suggested for all hospitalized medical cancer patients. DOACs were recommended for VTE prophylaxis in outpatients undergoing chemotherapy at intermediate or high risk of VTE with low gastrointestinal (GI) tract bleeding risk as an alternative to LMWH. Results from head-to-head clinical trials that compared DOACs with LMWH were summarized, along with new evidence for the treatment and prophylaxis of VTE in patients with cancer. Helpful charts that summarized the use and dosage of different types of DOACs were presented (Supplementary Table S5). Fondaparinux could be safely used in prophylaxis or during therapy, even if there were insufficient data to support its use as an alternative to LMWH. Mechanical methods were illustrated and analyzed in the guideline and suggested as a combined therapy. Prophylaxis was not routinely indicated for CVC patients, and no recommendations were given about inferior vena cava filter insertion.

### 3.4. The Italian Association of Medical Oncology (AIOM)

The AIOM guidelines were drafted in 2004 and revised in 2019. The authors focused on the following six topics: VTE and occult cancer, VTE prophylaxis in cancer surgery, VTE prophylaxis during chemotherapy or hormone therapy, VTE prophylaxis and central venous catheters (CVCs), treatment of VTE in cancer patients, and anticoagulation and prognosis of patients with cancer.

Prophylaxis with LMWH or UFH for at least 4 weeks was recommended for cancer patients undergoing major surgery. The authors emphasized the need for LMWH high dosage in oncological surgical patients. In hospitalized cancer patients, VTE prophylaxis with LMWH was highly recommended. Routine prophylaxis for patients with advanced cancer treated with chemotherapy was not suggested, even though it was proposed in subjects with additional risk factors. In these patients, DOACs could be safely adopted for prophylaxis as an alternative to LMWH. The Khorana score was directed to assess each patient’s VTE risk [34] (Table 3). Routine prophylaxis was not indicated for cancer patients undergoing CVC. The inferior vena cava filter was recommended in case of progression during anticoagulant treatment. Occult cancer screening in the case of idiopathic VTE was not recommended.

### 3.5. Spanish Society of Medical Oncology (SEOM) Clinical Guideline of Venous Thromboembolism and Cancer

Ten oncologists of the Society of Medical Oncology’s Cancer and Thrombosis Section developed the SEOM guidelines. In the prophylaxis section, they identified three groups of patients, hospitalized cancer patients, surgical patients, and ambulatory patients, giving practical recommendations based on risk factors. They suggested stratifying a patient’s VTE risk based on major risk assessment scores, such as the Khorana score [34] (Table 3) and ONKOTEV score [36] (Table 5). According to the authors, VTE prophylaxis with LMWH was suggested for all hospitalized patients recovering from acute disease and surgical oncological patients for 4 weeks. In the surgical setting, pharmacological prophylaxis should be started preoperatively, and mechanical methods should be added in combined therapy or used as monotherapy in case of contraindications to prophylactic drugs. Ambulatory patients undergoing systemic therapy at risk of VTE were suggested to be cured with LMWH or DOACs for at least 12 weeks. CVC patients should not be routinely treated with VTE prophylaxis; a CVC should be inserted on the right side. DOACs were advised as an alternative to LMWH if contraindicated in ambulatory outpatients requiring VTE prophylaxis.

### 3.6. European Society of Medical Oncology (ESMO)

The ESMO guidelines mainly concentrated on the evaluation of VTE risk factors in oncological patients. The authors eventually proposed using the Khorana score [34] (Table 3) to assess each patient’s risk. They recommended prophylaxis with LMWH or UFH to patients undergoing major cancer surgery and hospitalized patients only if bedridden or recovered from an acute medical illness. Mechanical methods such as a pneumatic compression calf could be added to pharmacological prophylaxis. Pneumatic compression should not be used as a monotherapy unless pharmacological prophylaxis was contraindicated due to active bleeding. Since the guidelines were published in 2011, there have not been safe indications concerning the use of DOACs in cancer patients.

### 3.7. The Haemostasis and Thrombosis Task Force of the British Committee for Standards in Haematology (BCSH)

The guideline was drafted by a writing group identified by the Haemostasis and Thrombosis Task Force of the British Committee for Standards in Haematology (BCSH) through a systematic review of the literature. The guideline was then reviewed by the sounding board of the British Society for Haematology (BSH).

The guideline strongly suggested VTE prophylaxis in all inpatients and only in outpatients with a Khorana score >2. During chemotherapy, VTE prophylaxis was indicated only in patients with a previous history of VTE. VTE prophylaxis was not recommended in hormonal replacement therapy. There was no suggestion about the duration of the prophylaxis. DOACs were proposed in prophylactic settings.

### 3.8. The Canadian Consensus Recommendation on the Treatment of VTE in Cancer Patients

This guideline was based on consensus and evidence on the topic, realized after a systematic literature review of clinical trials and meta-analysis published between 2002 and 2013. National Canadian guidelines on the prevention of cancer-associated thrombosis have not yet been published. The consensus suggested VTE prophylaxis in all surgical inpatients and only in outpatients with a Khorana score > 2 (Table 3). DOACs were only cited.

For medical inpatients, the authors recommended using VTE prophylaxis, basing this indication on data proving that the cancer population was at twice the risk of VTE during hospitalization than the general population.

### 3.9. Italian Society for Haemostasis and Thrombosis (SISET)

The SISET guidelines’ recommendations were formulated and graded according to the supporting evidence. A formal consensus method was used to issue clinical recommendations where no literature evidence was found. Pharmacological or mechanical prevention in cancer patients undergoing major abdominal or pelvic surgery was proposed using LMWH agents or fondaparinux. The prophylaxis should be started before surgery and extended for at least 7 days, up to 4 weeks if necessary. VTE medical prevention was also recommended in bedridden cancer patient who needed to be hospitalized for acute events. Prevention was not suggested for cancer patients with a CVC and those on systemic therapy.

### 3.10. The American Society of Hematology (ASH)

These guidelines are based on updated and original systematic reviews of evidence. The Grading of Recommendations, Assessment, Development, and Evaluation (GRADE) approach was used to assess evidence and make recommendations.

The ASH guideline panel suggests pharmacological thromboprophylaxis for hospitalized medical patients with cancer (LMWH or UFH if several renal impairments), for patients at low bleeding risk undergoing a surgical procedure (LMWH or fondaparinux), and ambulatory patients receiving systemic therapy at intermediate and high risk for thrombosis (DOAC as apixaban or rivaroxaban).

The mechanical thromboprophylaxis as monotherapy is considered for all patients at high risk for major bleeding either if hospitalized or undergoing a surgical procedure. A combination of pharmacological and mechanical prophylaxis may also be considered for selected hospitalized medical patients who are considered at very high risk for VTE (e.g., patients with cancer with sustained and prolonged immobilization).

For patients with cancer and a CVC, the ASH guideline panel suggests not using parenteral or oral thromboprophylaxis.

## 4. Discussion

We conducted a systematic review of the literature concerning guidelines on VTE prevention for gynecological cancer patients, and we found 10 eligible guidelines discussing the topic. Based on the results found in the literature, VTE prophylaxis in gynecological cancer patients is classified into three leading groups of patients:

- Hospitalized medical patients;
- Hospitalized surgical patients, preoperative and postoperative;
- Ambulatory outpatients on systemic therapy or undergoing CVC.

### 4.1. Prevention of VTE in the Hospitalized Medical Patient with Cancer

As previously described, hospitalized cancer patients are at a higher risk of VTE, twice that of the general population [11,12], with an increased specific risk in gynecological cancer patients [13,16]. The available prospective data [37,38] on VTE prevention in hospitalized patients are based on a mixed group of oncological and non-oncological patients; therefore, the risk varied significantly between different subgroups of subjects affected. However, almost all the guidelines listed above suggested starting VTE prophylaxis in all hospitalized inpatients.

ESMO guidelines focused on immobility as a crucial risk factor and recommended prophylaxis only in immobilized hospitalized cancer patients suffering from an acute illness.

The Canadian consensus concluded by recommending VTE medical prophylaxis for all hospitalized medical cancer patients, highlighting the lack of data on VTE prevention in oncological patients.

Finally, the NCCN [24], ASCO [25], AIOM [27], and ASH [33] guidelines strongly suggested beginning VTE medical prophylaxis for all hospitalized cancer patients; additionally, they proposed considering each patient’s risk once discharged from recovery and, based on the Khorana score, that VTE prophylaxis should be continued in at-risk medical patients even after discharge for up to 3–6 months using DOACs.

Interestingly, the ITAC/ISTH guidelines did not recommend VTE prophylaxis in medically hospitalized cancer patients, not specifying why.

### 4.2. Prevention of VTE in the Surgical Patient with Cancer

In oncological surgery, several studies suggested that LMWH had efficacy equal to UFH in perioperative prophylaxis [39,40,41].

All the guidelines mentioned above recommended prophylactic anticoagulation in surgical oncological settings. Before surgery, patients should receive pharmacological thromboprophylaxis with either low-dose UFH or LMWH or fondaparinux unless contraindicated (the off-label use of fondaparinux for intra- and perioperative anticoagulation in patients with heparin-induced thrombocytopenia seems to be possible) [42,43]. The duration of therapy was indicated in most of the guidelines. It should last for 7 days for minor surgery (less than 30 min procedures) and up to 4 weeks for major surgical procedures.

All the analyzed guidelines support only the use of LMWH or fondaparinux for VTE postoperative prophylaxis in oncological patients, even if a recent RCT has demonstrated the safety and efficacy of using DOACs in postoperative gynecological cancer patients but still not routinely being used in clinical practice [44,45].

Mechanical methods such as anti-embolic stockings and intermittent pneumatic compression were only cited in the most recent guidelines. The SEOM [28], AIOM [27], ASCO [25], and ASH [33] guidelines adopted mechanical methods as a monotherapy if pharmacological methods were contraindicated. Instead, for ITA/ISTH [26] guidelines, mechanical methods could be added to pharmacological methods but should not be adopted as a monotherapy for VTE prevention.

Perioperative intermittent pneumatic compression (IPC) devices are a non-negligible support in VTE prophylaxis. In a systematic review and meta-analysis by Jian Ping Feng et al. in 2017 [46] that considered seven randomized, controlled trials involving 1001 participants, IPC effectively reduced VTE complications in gynecologic surgery. The authors concluded by stating that IPC was neither superior nor inferior to pharmacological thromboprophylaxis.

Gynecological cancer patients are a particular subgroup of patients due to the high risk of bleeding from gynecological malignancies, the high demolition surgery they undergo, and the vascular risk of most surgical procedures, such as lymph node dissection, which increase the chances of postoperative bleeding.

Gynecological cancer patients in postoperative settings may represent a different category of oncological surgical patients who could benefit from a combination of mechanical and medical prophylaxis or IPC alone in particular cases where bleeding risk and thrombosis risk are equally very high.

### 4.3. Prevention of VTE in the Ambulatory Patient Undergoing Systemic Therapy

Chemotherapy may increase the risk of thromboembolism through at least three mechanisms:

- Acute damage on a vessel’s wall;
- Vessels’ delayed endothelial integrity damage; and
- The reduction of coagulation processes’ regulatory proteins, such as decreased protein C and S levels, i.e., reducing the antithrombin III (ATIII) level.

A considerable American study indicated that 12.6% of patients experienced VTE 12 months after starting chemotherapy [14,47].

Platinum-based therapy was indicated to induce platelet activation. Therefore, gynecological cancer patients who were more likely to undergo these therapies have a higher risk of VTE complications [48].

The various guidelines analyzed broadly agreed on not recommending routine thromboprophylaxis because the VTE incidence in outpatients receiving chemotherapy was still low (1–5%) and potentially exposed the patient to a bleeding risk.

All the guidelines mentioned above suggested medical prophylaxis in ambulatory patients under systemic therapy only if considered at a high risk based on Khorana or other VTE risk scores.

The use of DOACs was established and demonstrated to be safe in this subset of patients; therefore, the most recent guidelines introduced it.

In CVC patients, medical prophylaxis was not routinely indicated; some of the guidelines listed suggested inserting a CVC on the right side and preferably using a port instead of peripherally inserted central venous catheters (PICC) to reduce CVC–VTE-related complications.

Regarding medical prophylaxis, all the authors agreed on the better effects of LMWH, UFH, or fondaparinux in preventing VTE in cancer patients than oral anticoagulants. Only the most recent ones recommended the safe use of DOACs in prophylaxis instead of LMWH in outpatients considered to be at a high risk of VTE. Other DOACs such as dabigatran (a direct thrombin inhibitor) have been studied only in treatment settings and, therefore, were not included in the analyzed guidelines [49].

Concerning mechanical prophylaxis, even if all the guidelines strongly suggested mechanical prophylactic methods, only the NCCN guidelines specified the type of mechanical compression to be used as a monotherapy (IPC, intermittent pneumatic compression) where contraindications to medical treatment were present, underlining the importance of considering the high risk of VTE in each patient. Indeed, gynecological oncological patients were also at a high risk of bleeding due to the intrinsic nature of the tumors, such as endometrial, myometrial, and cervical malignancies in advanced stages. Moreover, gynecological oncological surgical procedures, such as cytoreductive debulking and lymph node dissection, expose the patient to a higher postoperative bleeding risk; the balancing of bleeding versus VTE risk may be complex. In this subset of patients, IPC should be considered to balance the bleeding and thrombotic risk for each subject.

Furthermore, although radiotherapy and chemotherapy have been shown to increase the risk of VTE [49], the subgroup of patients under radiotherapy is not described in the guidelines analyzed. Patients undergoing radiotherapy as well as outpatients receiving chemotherapy should be risk stratified and given VTE prophylaxis when indicated.

Finally, newly diagnosed gynecological cancer patients waiting for the first primary surgical procedure, such as individuals affected by ovarian cancer in advanced stages, were not included in the presented guidelines. We believed this subgroup of patients requires specific attention because the extended and diffused carcinosis and ascites could aggravate an already present predisposition to VTE, exposing patient to these complications before surgery and treatment even if not already hospitalized. Therefore, we suggested methodically applying the risk stratification to all oncological gynecological patients from the moment of diagnosis to prevent VTE before preoperative assessment.

There are some limitations of this study. The numbers of studies included was limited and we did not perform any statistical analysis, limiting the value of the data exposed. Strengths of this work are to be considered. First of all, we compared the most recent recommendations given by national and international societies concerning the topic. The latest updates on medical prophylaxis have been highlighted. Lacking fields such as systematically stratifying each patient’s VTE and bleeding risk from the moment of diagnosis were pointed out during the review.

## 5. Conclusions

Despite convincing data and increased awareness by clinicians, there is still significant heterogeneity in daily clinical practice for prophylactic protocols of VTE in oncological patients. All the guidelines mentioned above suggested using a VTE risk scoring system to stratify each patient’s risk, whether surgical or medical inpatients or ambulatory patients receiving chemotherapy. Almost all the guidelines quoted the Khorana score (Table 3). The most recent guidelines (ASCO and NCCN) also suggested evaluating the risk of each patient considered at the moment of discharge and continuing medical prophylaxis of those patients at high risk [24,25]. Only the most recent guidelines emphasized the importance of VTE prevention in each patient, suggesting employing DOACs in outpatients undergoing chemotherapy and considered at high risk. This allows the adherence of these subjects to the therapy and extends the prophylaxis also to the discharged. Mechanical VTE prophylactic methods are to be considered in patients at high risk of bleeding. Further effort should be made to create an algorithm to standardize the timing and prophylaxis for VTE in cancer patients.

## Figures and Tables

**Table 1:** AGREE II domain and total score of clinical practice guidelines included in percentage and absolute numbers.

Cpg	Scope and Purpose	Stakeholder Involvement	Rigor of Development	Clarity of Presentation	Applicability	Editorial Independence	Overall Assessment
**NCCN 2020**	100 (21/21)	100 (21/21)	100 (56/56)	100 (21/21)	100 (28/28)	100 (14/14)	100 (7/7)
**ASCO 2020**	100 (21/21)	100 (21/21)	1000 (56/56)	100 (21/21)	100 (28/28)	100 (14/14)	100 (7/7)
**ITAC/ISTH 2019**	100 (21/21)	100 (21/21)	100 (56/56)	100 (21/21)	100 (28/28)	100 (14/14)	100 (7/7)
**AIOM 2019**	100 (21/21)	100 (21/21)	96.4 (54/56)	100 (21/21)	100 (28/28)	92.8 (13/14)	100 (7/7)
**SEOM 2019**	95.2 (20/21)	90.4 (19/21)	94.6 (53/56)	95.2 (20/21)	82.1 (23/28)	100 (14/14)	85.7 (6/7)
**BCSH 2015**	90.4 (19/21)	76.1 (16/21)	87.5 (49/56)	100 (21/21)	92.8 (26/28)	100 (14/14)	85.7 (6/7)
**Canadian Consensus 2015**	90.4 (19/21)	90.4 (19/21)	87.5 (49/56)	95.2 (20/21)	85.7 (24/28)	92.8 (13/14)	71.4 (5/7)
**ESMO 2011**	100 (21/21)	95.2 (20/21)	91.1 (51/56)	100 (21/21)	100 (28/28)	85.7 (12/14)	85.7 (6/7)
**SISET 2011**	100 (21/21)	95.2 (20/21)	85.7 (49/56)	80.9 (17/21)	89.2 (25/28)	85.7 (12/14)	71.4 (5/7)
**ASH 2021**	100 (21/21)	100(21/21)	100 (56/56)	95.2(20/21)	100(28/28)	92.8(13/14)	100(7/7)

**Table 2:** VTE prevention in cancer patients: guidelines’ recommendations.

Guideline	Prevention in Hospitalized Medical Patients	Prevention in Surgical Patients	Timing and Duration	Prevention in Ambulatory Patients under Systemic Therapy	Prevention in CVC Cancer Patients
**NCCN 2020**	LMWH or UFH or fondaparinux in inpatients: DOACs (apixaban or rivaroxaban) after discharge in all hospitalized gynecological cancer patients, up to 6 months if Khorana score >2 after discharge	LMWH, UFH, and fondaparinux in major abdominal or pelvic surgery	for 30 days after admission	DOACs (apixaban or rivaroxaban) up to 6 months only indicated in high-risk patients (Khorana score)	not indicated as routine prophylaxis
**ASCO 2020**	LMWH or UFH or fondaparinux in all hospitalized gynecological cancer patients and after discharge in high-risk patients (Khorana score)	LMWH: Mechanical methods can help prophylaxis but should be used as monotherapy only if medical prophylaxis contraindicated. Fondaparinux or UFH can be considered in major cancer surgery (pelvic, abdominal).	should be started preoperatively, last for 7 days, and extended to 4 weeks after major procedures	LMWH or DOACs (apixaban or rivaroxaban) only indicated in high-risk patients (Khorana Score)	\
**ITAC/ISTH 2019**	LMWH or fondaparinux in gynecological cancer patients with reduced mobility	LMWH or UFH: Mechanical methods should not be used as monotherapy; in case of major laparotomy and all laparoscopies, should be started preoperatively	for 30 days after admission	DOACs (apixaban or rivaroxaban or edoxaban) only indicated high-risk patients (Khorana Score, COMPASS-KAT score, ONKOTEV score)	not indicated as routine prophylaxis; should be inserted on the right side and use PORT instead of PICC
**AIOM 2019**	LMWH or UFH or fondaparinux in all hospitalized gynecological cancer patients	LMWH: Mechanical methods suggested but should be used as monotherapy only if prophylaxis is contraindicated. Fondaparinux or UFH can be considered in major cancer surgery (pelvic, abdominal)	should be started preoperatively, last for 7 days, and extended to 4 weeks in high-risk groups	LMWH or DOACs (apixaban or rivaroxaban) only indicated in high-risk patients (Khorana Score)	not indicated as routine prophylaxis
**SEOM 2019**	LMWH in gynecological cancer patients with concomitant acute medical illness	LMWH and mechanical or only mechanical if LMWH contraindicated in all surgical interventions, should be started preoperatively	should be started preoperatively, last for at least 7 days, and extended to 4 weeks in high-risk patients	LMWH or DOACs for 12 weeks after initiation therapy only indicated in high-risk patients (Khorana or other models suggested)	not indicated as routine prophylaxis
**ESMO 2011**	LMWH or UFH or fondaparinux in gynecological cancer patients with reduced mobility or acute medical illness	UFH or LMWH in all surgical gynecological cancer patients	for 30 days after admission	LMWH indicated only in high-risk patients	not indicated as routine prophylaxis
**BCSH 2015**	LMWH in all inpatient gynecological cancer patient	in case of major abdominal or pelvic surgery	\	LMWH or warfarin only indicated in high-risk patients (Khorana Score); not indicated in hormonal replacement therapy if no previous VTE	not indicated as routine prophylaxis
**Canadian Consensus 2015**	LMWH in all inpatient gynecological cancer patients	all surgical cancer patients	for 30 days after admission	LMWH only indicated in high-risk patients (Khorana Score>2)	not indicated as routine prophylaxis
**SISET 2011**	LMWH of fondaparinux: mechanical prophylaxis if high risk of bleeding or anticoagulant contraindications in gynecological cancer patient with concomitant acute medical illness	LMWH, UFH, and fondaparinux in all surgical gynecological cancer patients	should be started preoperatively, last for 7 days, and extended to 4 weeks in high-risk patients	not indicated	not indicated as routine prophylaxis
**ASH 2021**	LMWH (UFH) in patients with severe renal impairment, mechanical prophylaxis if high risk for bleeding, mechanical and pharmacological prophylaxis if very high risk for TVE	LMWH or fondaparinux for patients at low bleeding risk, mechanical prophylaxis as monotherapy if LMWH contraindicated, combination of pharmacological and mechanical methods for patients at high risk of thrombosis	Should be started postoperatively, extended up to 4 weeks	LWHM only for patients receiving systemic therapy at intermediate and high risk for thrombosis (Khorana score complemented by clinical judgment and experience)	not indicated as routine prophylaxis

**Table 3:** Khorana score VTE risk assessment predictive model in cancer outpatients.

Patient Chacteristic	Risk Score
Site of primary cancer	
Very high risk (stomach, pancreas)	2
High risk (lung, lymphoma, gynecologic, bladder, testicular)	1
Prechemotherapy platelet count 350 × 10^9^/L or higher	1
Hemoglobin level less than 10 g/dL or use of red cells’ growth factors	1
Prechemotherapy leukocyte count higher than 11 × 10^9^/L	1

**Table 4:** COMPASS-KAT VTE risk score assessment.

Predictors for VTE	Score
Cancer-related risk factors	
- Anti-hormonal therapy for women with hormone receptor-positive breast cancer or on anthracycline treatment	6
- Time since cancer diagnosis < 6 months	4
- CVC	3
- Advanced stage cancer	2
Predisposing risk factors	
- Cardiovascular risk factors (composed of at least two of the following predictors: personal history of peripheral artery disease, ischemic stroke, coronary artery disease, hypertension, hyperlipidemia, diabetes, obesity)	5
Recent hospitalization for acute medical illness	5
Personal history of VTE	1
Biomarkers	
Platelets count > 350 × 10^9^/L	2

Low/Intermediate risk: 0–6; high risk: >7.

**Table 5:** ONKOTEV VTE risk score assessment.

Risk Factor	Score
Khorana score > 2	1
Previous thromboembolism	1
Metastatic disease	1
Vascular/lymphatic macroscopic compression	1
Total ONKOTEV score	4

## Data Availability

The data presented in this study are available in the article and tables.

## Supplementary Materials

The following supporting information can be downloaded at: https://www.mdpi.com/article/10.3390/cancers14102439/s1, Table S1: NCCN VTE risk factors in patients with cancer; Table S2: NCCN contraindication to VTE prophylaxis; Table S3: NCCN prophylactic anticoagulan option for inpatients and surgical pa-tients with cancer; Table S4: NCCN warnings and contraindications for the use of anticoagulant drugs; Table S5: Characteristics of direct anticoagulants in patients with cancer, from ITAC/ISTH guidelines 2019.

## Abbreviations

VTE	Venous thromboembolic events
CVC	Central venous catheter
UFH	Unfractionated heparin
LMWH	Low-molecular-weight heparin
IPC	Intermittent pneumatic compression
DOACs	Direct oral anticoagulants
MMX	Mammography
PICC	Peripherally inserted central catheters
PORT	Centrally inserted totally implanted vascular access
