# Supplementary material for: A Systematic Review of the Guidelines on Venous Thromboembolism Prophylaxis in Gynecologic Oncology
Source: Cancers (Basel). 2022 May 15;14(10):2439. doi: 10.3390/cancers14102439 (PMC9139174; doi:10.3390/cancers14102439)
Supplement: Supplementary file 1 [file cancers-14-02439-s001.zip › cancers-1687015-supplementary.pdf]

SUPPLEMENTARY

Table S1 – NCCN VTE risk factors in patients with cancer

General patient risk factors
Active cancer
Advanced stage of cancer
Cancer types at higher risk:
Brain
Pancreas
Stomach
Bladder
Gynecologic
Lung
Lymphoma
Myeloproliferative neoplasms (MPN)
Kidney
Metastatic cancers
Regional bulky lymphoadenopathy with extrinsic vascular compression
Familiar and acquired hypercoagulability (including pregnancy)
Medical comorbidities: infection, renal disease, pulmonary disease, congestive heart failure (VHF), arterial thromboembolism
Poor performance status
Older age
**Modifiable risk factors**
Smoking, tobacco
Obesity
Activity level/excercise

High-risk outpatients on chemotherapy, based on combinations of the following risk factors
Active cancers associated with high incidence of VTE: stomach, pancreas, lung, lymphoma, gynecologic, bladder, testicular
Prechemotherapy platelet count>350.000/mcL
Prechemotherapy white blood cell(WBC) count>11.000/mcL
Hemoglobin<10g/dL
Use of erythropoiesis-stimulating agents (ESAs)
BMI 35 or greater
Prior VTE
**Treatment related-risk factors**
Major surgery
Central venous catheter/IV catheter
Chemotherapy such as:
IMiDs plus high-dose dexamethasone
Proteasome inhibitors
Exogenous hormonal therapies such as
Hormone replacement therapy (HRT)
Contraceptives
Tamoxifene/raloxifene
Diethylstilbestrol

Table S2 – NCCN contraindication to VTE prophylaxis

Contraindications to Prophylactic Anticoagulation
Active bleeding
Thrombocytopenia (platelets <30.000-50.000/mcL or clinical judgement)
Underlying hemorragic coagulopaty (eg. Abnormal PT or aPTT excluding a lupus inhibitor/anticoagulant) or known bleeding disorder in the abscence of replacement therapy (eg. Hemophilia, von Willebrand disease)
Indwelling neuraxial catheters (contraindication for apixaban, dabigatran, edoxaban, fondaparinux, rivaroxaban, or enoxaparin dose exceeding 40 mg daily)
Neuraxial anesthesia/lumbar puncture
Interventional spine and pain procedures

Contraindications to Mechanical Prophylaxis
Absolute
Acute DVT
Severe arterial inssuficiency (pertains to graduate compression stockings only)
Relative
Large hematoma
Skin ulcerations or wounds
Thrombocytopenia (platelets < 20.000/mcL)
Mild arterial insufficiency (pertains to GCS only)
Peripheral neuropathy (pertains to GCS only)

Table S3 – NCCN prophylactic anticoagulan option for inpatients and surgical patients with cancer

Agent	Standard Dosing	Obesity Dosing (BMI >40)
LMWH: Dalteparin	5,000 units SC daily	Consider 7,500 units SC daily (limited data)
LMWH: Enoxaparin	40 mg SC daily	Consider 40 mg SC every 12 hours
Fondaparinux	2.5 mg SC daily	Consider 5 mg SC daily (limited data)
UFH	5,000 units every 8-12 hours	Consider 7,500 units SC every 8 hours

Table S4 – NCCN warnings and contraindications for the use of anticoagulant drugs

Agent(s)	Contraindications and Warnings
LMWH	- use with caution in patients with renal dysfunction; consider dose adjustments or alternative therapy for patients with severe renal disfunction (CrCl <30 mL/min)<br>- follow package insert for renal dysfunction and body weight dosing<br>- Anti-Xa monitoring (peak and trough) of LMWH has been. Reccomended for patinets with severe renal dysfunction, althoug limited data are available to support the clinical relevance of anti-Xa levels<br>- Absolute contraindication: recent/acute HIT<br>- Relative contraindication: past history of HIT
Fondaparinux	- Contraindicated in patinets with CrCl <30 mL/min<br>- Use with caution in patients with moderate renal insufficiency (CrCl 30-50 mL/min), weight <50 Kg or age >75)
UFH	- Absolute contraindication: recent/acute HIT<br>- Relative contraindication: past history of HIT
Warfarin	Relative contraindications: concomitant inhibitors and inducers of CYP2C9, 1A2 or 3A4
DOACs: Apixaban, dabigatran, edoxaban and rivaroxaban	Contraindications:<br>- Stage IV/V chronic kidney disease<br>- Apixaban: CrCl <25mL/min<br>- Dabigatran, edoxaban and rivaroxaban: CrCl<30mL/min<br>- Active/clinically significant liver disease:<br>- Apixaban or edoxaban: ALT/AST>2xULN; total bilirubin>1.5xULN<br>- Dabigatran or rivaroxaban: ALT/AST>3xULN<br>- Strong dual inhibitors/inducers of CYP3A4 and P-glycoprotein (P-gp): see prescribing information for rivaroxaban and apixaban<br>- Inducers/inhibitors of P-gp: see prescribing information for dabigatran and edoxaban<br>Relative contraindications, use with caution:<br>- DOACs have bee associated with an increased risk of gastrointestinal and possibly genitourinary tract bleeding, and should be used with caution in patients with genitourinary or gastrointestinal tract lesions, pathology or instrumentation<br>- Use with caution in patients with compromised renal or liver function<br>- For patinets receiving nephrotoxic or hepatotoxic chemotherapy consider monitoring patients more closely with laboratory testing<br>- Consider drug-drug interactions

Table S5 - Characteristics of direct anticoagulants in patients with cancer, from ITAC/ISTH guidelines 2019

	**Dabigatran**	**Rivaroxaban**	**Apixaban**	**Edoxaban**
**Target**	FIIa	FXa	FXa	FXa
**Dosing**	Therapeutic :150mg twice daily; 110mg twice daily for patients>80 years following at least days of parenteral anticoagulant	Therpeutic: 15mg twice daily for 3 weeks followed by 20mg once daily	Therapeutic: 10mg daily for 7 days, followed by 5mg twice daily	Therapeutic: 60mg once daily following at least 5 days of parenteral anticoagulants
**Prodrug**	Yes	No	No	no
**Bioavailability**	3-7%	10mg dose: 100%; 20mg dose 100% when taken together with food, 66% under fasting conditions, inter-individual variability: 30-40%	-50%; interindividual variability: 30%	-62%
**Activity onset**	1-3 h	2-4 h	3-4 h	1-2 h
**Half-life**	12-18 h	5-13 h	12 h	10-14 h
**Excretion (% of administer dose)**	80% renal (unchanged), 20% liver	66% renal (half active drug unchanged and half inactive metabolites), 33% faeces (inactive metabolites)	25% renal, 75% faeces	50% renal (unchanged), 50% biliary or intestinal
**Considerations for hepatic insufficiency**	Liver enzymes twice normal limit or if acute liver disease: not reccomended	Moderate hepatic impairment: caution required, hepatic disease with coagulopathy and clinically relevant bleeding risk: contraindicated	Mild or moderate hepatic impairment: caution, but no dose adjustment required; severe hepatic impairment: not reccomended; hepatic disease with coagulopaty and clinically relevant bleeding risk: contraindicated	Mild hepatic impairment: no dose reduction; moderate or severe hepatic impairment: not reccomended
**Interaction**	P-glycoprotein inducers or inhibitors	P-glycoprotein inducers or inhibitors, CYP3A4, CYP2J2	P-glycoprotein inducers or inhibitors, CYP3A4	P-glycoprotein inducers or inhibitors, CYP3A4
**Specific trials in patients with cancer**	None	SELECT D, CASSINI	ADAM-VTE, AVERT	HOKUSAI
**Specific antidote**	Idarucizumab, aripazine	Andexanet alfa, aripazine	Andexanet alfa, aripazine	Andexanet alfa, aripazine
